# Oral bisphosphonates and colorectal cancer

**DOI:** 10.1038/srep44177

**Published:** 2017-03-10

**Authors:** Emily Vogtmann, Douglas A. Corley, Lucy M. Almers, Chris R. Cardwell, Liam J. Murray, Christian C. Abnet

**Affiliations:** 1 Metabolic Epidemiology Branch, Division of Cancer Epidemiology and Genetics, National Cancer Institute, National Institutes of Health, Bethesda, Maryland, USA; 2 Kaiser Permanente, San Francisco Medical Center, San Francisco, California, USA; 3 Centre for Public Health, Queen’s University Belfast, Belfast, United Kingdom

## Abstract

Use of oral bisphosphonates has been associated with a decreased risk of colorectal cancer (CRC), but the association may be related to residual confounding by healthy lifestyle or body mass index (BMI). Therefore, we conducted a prospective nested case-control study within the Kaiser Permanente, Northern California health system cohort. In total, 12,505 CRC cases were individually matched to 599,534 controls. Odds ratios (OR) and 95% confidence intervals (CI) were calculated using conditional logistic regression models with adjustment for important covariates extracted from the database. Participants who had ever used oral bisphosphonates were less likely than non-users to be diagnosed with CRC (OR 0.82; 95% CI: 0.74, 0.89). Colon and rectum site-specific associations were similar to the overall association. A stronger inverse association for ever use of bisphosphonates was observed for men (OR 0.63; 95% CI: 0.47, 0.85), however when stratified by previous lower endoscopy, the association was only observed in the participants who did not have a previous lower endoscopy (OR 0.73 (0.64, 0.83)). In conclusion, we found that oral bisphosphonate use was associated with a decreased odds of CRC, however this association may be due to residual confounding by BMI or another confounder.

Osteoporosis is a highly prevalent condition within the aging population of the United States, where it is estimated that 10.3% of the population aged 50 and older have osteoporosis^1^. Bisphosphonates are widely prescribed drugs used for the treatment of osteoporosis, so exposure to bisphosphonates is common among older adults. There is some evidence that bisphosphonates, in addition to inhibiting bone resorption, may also have anti-tumorigenic effects through several proposed mechanisms^2^. One proposed mechanism is through human epidermal growth factor receptors (HER) where nitrogen containing bisphosphates appear to kill specific cancer cell lines, including colon cancer cell lines, which either have activating mutations or overexpression of HER1^3^.

Some previous studies have considered the association between oral bisphosphonate use and the risk of colorectal cancer^4^,^5^,^6^,^7^,^8^,^9^,^10^,^11^,^12^,^13^. These studies generally found an inverse association (i.e., a decreased risk of colorectal cancer with use of oral bisphosphonates)^4^,^5^,^6^,^9^,^10^ or no significant association^7^,^8^,^11^,^12^,^13^. With the exception of the earliest meta-analysis^14^, the resulting meta-analyses have concluded that oral bisphosphonates are associated with a decreased risk of colorectal cancer^15^,^16^,^17^,^18^,^19^. However, a recent review of observational studies and meta-analyses considering the association of oral bisphosphonates and colorectal cancer suggested that residual confounding may explain the detected inverse associations^20^. Additionally, only a few studies^5^,^6^,^10^ considered site-specific associations. Colon and rectal cancer do not share all of the same risk factors and therefore, the association may be different by site. To address these issues, we conducted a nested case-control study considering how oral bisphosphonate use was associated with the risk of colon and rectal cancer using data from the Kaiser Permanente, Northern California (KPNC) health system.

## Results

From 1997 to 2011 in KPNC there were 12,505 colorectal cancer cases which were matched by sex, age at time of index date (+/−2 years), duration of membership prior to the index date (+/−1 year), race, and region of residence to 599,534 controls. Membership duration prior to the index date ranged from a minimum of 2 years to a maximum of 15.4 years with an interquartile range of 4.6 to 11 years. A total of 563 colorectal cancer cases (4.50%) had ever used oral bisphosphonates at least one year prior to the index date compared to 32,897 (5.49%) of the controls. The colorectal cancer cases had a higher proportion of smokers (40.09%) compared to the controls (35.84%) and a higher proportion of alcohol consumers (24.77% versus 21.94%) (Table 1).

Overall, participants who had ever used bisphosphonates at least one year prior to the index date were less likely to develop colorectal cancer than non-users (odds ratio (OR) 0.77; 95% confidence interval (CI): 0.71, 0.85) in unadjusted conditional logistic regression models. After adjustment for age, smoking, alcohol use, the Charlson comorbidity index (CCI), use of non-steroidal anti-inflammatory drugs (NSAIDS), and previous lower endoscopy, the OR was slightly attenuated (OR 0.82; 95% CI: 0.74, 0.89). The OR were general similar for shorter (<12 months) and longer duration (≥12 months) of oral bisphosphonate use as measured using the Defined Daily Dose (DDD) (Table 2). When previous lower endoscopy was excluded from adjustment, the OR estimates were similar to the unadjusted models (Supplemental Table 1).

Oral bisphosphonate use was associated with a decreased odds of colorectal cancer for most cancer sites. For example, the ORs for cancer of the cecum, ascending colon, sigmoid colon, and rectum were 0.86 (95% CI: 0.72, 1.03), 0.79 (95% CI: 0.63, 0.99), 0.77 (95% CI: 0.61, 0.96), and 0.73 (95% CI: 0.56, 0.94) respectively (Table 3). Among the 51.32% of participants with body mass index (BMI) data, inclusion of BMI slightly attenuated the association between ever use of bisphosphonates and colorectal cancer from an OR of 0.89 (95% CI: 0.80, 0.99) to an OR of 0.92 (95% CI: 0.83, 1.02) (Table 4). Stratified models within normal weight, overweight, and obese individuals provided generally similar association estimates between bisphosphonates and colorectal cancer (Supplemental Table 2). Since participants who had measured BMI data in this cohort tended to have been a member of KPNC for more years (mean 10.2 years) compared to those without BMI data (mean 5.4 years), we stratified the sample by membership duration, less than or equal to the first quartile of membership duration (≤4.6 years) and the top three quartiles (>4.6 years). A stronger association was detected among participants with a short membership duration with an OR for ever use of oral bisphosphonates of 0.66 (95% CI: 0.48, 0.91), but the association within participants with a longer duration of membership was similar to the original model (Supplemental Table 1).

A stronger inverse association for ever use of bisphosphonates was observed for men (OR 0.63; 95% CI: 0.47, 0.85), although the interaction between sex and oral bisphosphonates on colorectal cancer was only statistically significant for the ever use of oral bisphosphonates (p = 0.0426) (Table 5). When we included adjustment for BMI in the stratified analysis, the estimates were only slightly attenuated compared to the model within the population with BMI measured (Supplemental Table 3). In the models for women, adjustment for hormone replacement therapy (HRT) use did not change the estimates (Supplemental Table 1).

When the data were stratified by having a previous lower endoscopy at least one year prior to the index date, the inverse association between ever use of oral bisphosphonates was only observed in the participants who did not have a previous lower endoscopy at least one year prior to the index date (OR 0.73; 95% CI: 0.64, 0.83), while no association (OR 0.92; 95% CI: 0.80, 1.04) was observed among those that had a previous lower endoscopy (Table 6).

## Discussion

In this study of 12,505 colorectal cancer cases and 599,534 controls from KPNC, we found that bisphosphonate use was associated with a decreased odds of colorectal cancer particularly among participants without prior endoscopic colorectal cancer screening procedures. In general, bisphosphonates decreased the odds of cancer in colon and rectum site-specific analyses. There was some evidence for residual confounding by BMI, although the association between bisphosphates and colorectal cancer was overall weaker within participants who had BMI measured compared to the whole study population. The association between bisphosphonate use and colorectal cancer was stronger within males compared to females and the association was restricted to participants who did not have a previous lower endoscopy at least one year prior to the index date.

Our overall finding of a decreased odds of colorectal cancer for participants who used oral bisphosphonate is in agreement with a number of previous studies^4^,^5^,^6^,^9^,^10^ and the majority of previous meta-analyses^15^,^16^,^17^,^18^,^19^. In a previous study which evaluated the association by site also found inverse associations between oral bisphosphonate use and cancer of the right colon (OR 0.49; 95% CI: 0.29, 0.85), left colon (OR 0.50; 95% CI: 0.29, 0.86), and rectum (OR 0.50; 95% CI: 0.23, 1.08)^5^. Another study found a stronger inverse association between bisphosphonates and cancer of the distal colon^6^ while a third study found a decreased risk for cancers of the colon and rectum, but not for cancers of the rectosigmoid junction^10^.

Of the studies which adjusted for BMI, three studies found an inverse or marginally inverse association^4^,^5^,^9^, while three other studies found no statistically significant association^11^,^13^ or a marginally weak increased risk^7^ of colorectal cancer after use of oral bisphosphonates. Three of these studies included overlapping populations from the Clinical Practice Research Datalink (CPRD; previously known as the General Practice Research Database)^4^,^7^,^9^, and the most recent study^7^ had the lowest percentage of missing BMI data and found a weakly positive association (OR 1.10; 95% CI: 1.00, 1.22) between bisphosphonate use and colorectal cancer specifically within the CPRD^7^. This suggests that the inverse association may be due to residual confounding by BMI^20^. We similarly found no significant association between oral bisphosphonates and colorectal cancer after adjustment for BMI, although a large proportion of participants were missing BMI data and the association between oral bisphosphonates and colorectal cancer was weaker within the population that had BMI data. This weaker association may be related to membership duration since participants with measured BMI tended to be members of KPNC for a longer duration, but when participants with a short duration of membership were excluded, the association between bisphosphonates and colorectal cancer was unchanged from the initial estimate. In addition, participants with a longer membership duration would have more accurate measures of bisphosphonate exposure compared to those with a shorter duration of membership.

Many previous studies were conducted only among women^5^,^10^,^11^,^12^,^13^. Of these, only two studies^5^,^10^ detected a decreased risk of colorectal cancer among women who used oral bisphosphonates. We detected a statistically significant inverse association between oral bisphosphonates and colorectal cancer among women, however a stronger inverse association was detected for men. In a nested case-control study within the Manitoba Health administrative database, a stronger, albeit not statistically significant association with colorectal cancer was detected within men (OR 0.56; 95% CI: 0.28, 1.11) compared to women (OR 0.81; 95% CI: 0.67, 0.98) for having 14 or more versus 0–1 bisphosphonate prescriptions^6^ which was very similar to our estimates for men (OR 0.56; 95% CI: 0.36, 0.86) and women (OR 0.83; 95% CI: 0.74, 0.94) for a defined daily dose of a year or more of oral bisphosphonates. However, in one of the CPRD studies, the association estimates were similar for men (hazard ratio [HR] 0.78; 95% CI: 0.47, 1.28) and women (HR 0.74; 95% CI: 0.56, 0.97)^9^. It has been suggested that bisphosphonate users may be a more health conscious group^20^, which may be particularly pronounced among men and may explain our stronger findings.

Few previous studies evaluated effect modification by previous lower endoscopy^11^,^13^. In one of these studies, no associations were detected between oral bisphosphonate use and colorectal cancer in both the group with a previous lower endoscopy (HR 1.08; 95% CI: 0.75, 1.54) and the group without a previous lower endoscopy (HR 1.03; 95% CI: 0.74, 1.43)^11^. In our data, the inverse association between oral bisphosphonates and colorectal cancer appeared to be restricted to participants who had not had a lower endoscopy at least one year prior to the index date. Another previous study found an association between oral bisphosphonates and colorectal cancer overall, but when the analyses were restricted to advanced stage colorectal cancer, no statistically significant association was detected^6^. This may suggest that oral bisphosphonates may only prevent slow growing or early stage cancers, which would not be detected when regular lower endoscopies are used since these slow growing cancers would be removed in a lower endoscopy. However, we were unable to consider cancer stage and therefore this hypothesis should be tested in an independent population with additional data. The effect of oral bisphosphonates may also be similar to that of aspirin on colorectal cancer risk. In a cost-effectiveness analysis of aspirin, it was found that the use of aspirin for chemoprevention would only be beneficial in a population without colorectal cancer screening since colorectal cancer screening is highly cost-effective and greatly reduces the risk of colorectal cancer^21^.

This study has some limitations. All of the data for this study were obtained from claims data which may not completely represent the participants’ exposure history. For example, BMI data were missing for almost half of the participants, so the analyses which adjusted for BMI were restricted to participants with recorded BMI data. In addition, we were unable to adjust for a number of other potential confounders such as supplemental calcium and vitamin D since these are commonly prescribed over the counter and therefore would not be included in claims data, although a recent trial found no utility of a combination of aspirin, calcitriol, and calcium carbonate to prevent colorectal adenoma recurrence^22^. We only had limited information about lower endoscopy, so we were unable to investigate whether these lower endoscopies found and removed polyps. This study also was conducted in an insured population, so our findings may not be representative of individuals without insurance or without comprehensive coverage. However, a previous study suggests that the KPNC population is fairly representative of the region of coverage^23^. Finally, we did not have information on cancer stage at diagnosis, so future studies should further consider the association between oral bisphosphonates and colorectal cancer stage.

There are also a number of strengths. Our study included a very large number of colorectal cancer cases and controls from a comprehensive insurance system in the United States, with comprehensive capture of pharmacy exposure data and cancer outcomes. Due to this large sample size, we were also able to look at detailed site-specific association. Since enrollees of KPNC receive almost all of their health care through KPNC, it is unlikely that there are significant missing utilization or prescription data. The cancer registry of KPNC captures over 98% of cancer diagnoses and all colorectal cancer cases were identified through this registry.

In conclusion, we found an inverse association between oral bisphosphonate use and colorectal cancer within participants who had not had a previous lower endoscopy, however this association may be due to residual confounding by BMI, healthy lifestyle, or another unmeasured confounder. It is unlikely that oral bisphosphonates should be used to decrease colorectal cancer risk at the population level.

## Methods

### Source population

For this study, we used similar methods to a previously published study of oral bisphosphonates and upper gastrointestinal cancer in KPNC^24^. We selected cases and controls from adults (≥18 years old) who were members of the KPNC health system from 1997 to 2011. Kaiser Permanente is the largest not for profit health plan in California with membership around 3.3 million members in northern California who have been found to be generally representative of the residents of that region^23^. The KPNC Institutional Review Board approved this study and the requirement of written informed consent was waived. All methods were performed in accordance with relevant guidelines and regulations.

### Case ascertainment

Cases of invasive colorectal cancer (ICD-10: C18, C19, and C20) were selected from the KPNC cancer registry and the index date was considered the date of the diagnosis. To be included, cases had to be at least 18 years old at the colorectal cancer diagnosis and have at least 2 years of membership in KPNC prior to diagnosis. If a case had a history of cancer prior to colorectal cancer (identified using the cancer registry or ICD-9 V codes) or a history of Paget’s disease (ICD-9: 731.0) that case was excluded. We identified 12,505 colorectal cancer cases in total. We assigned the specific site within the colon and rectum based on the ICD-O-3 codes.

### Control selection

We identified up to 50 controls in the KPNC database which were matched without replacement to the cases based on sex, age at the index date (+/−2 years), membership duration before the index date (+/−1 year), race, and residential region. To be included, controls had to be at least 18 years of age, a KPNC member at the time of the index date for the matched case, and been a KPNC member for at least two years before the matched case’s index date. We identified 599,534 controls for this study.

### Assessment of oral bisphosphonate exposure

We collected data on prescription fills of oral bisphosphonate, including alendronate, etidronate, ibandronate, risedronate, and tiludronate, for the time before the index date for both cases and controls. The exposure to oral bisphosphonates was categorized as ever exposed (at least one prescription fill one year of more before the index date) or never exposed. For the participants who were ever exposed to oral bisphosphonates, the defined daily dose (DDD) of bisphosphonate exposure was calculated for data collected before the index date. We dichotomized the DDD variable as less than 12 months exposed or 12 or more months exposed^25^.

### Other covariates of interest

Other covariates before the index date were utilized for analysis. We included age, sex, race/ethnicity, history of smoking and alcohol consumption, BMI, the CCI^26^, a diagnosis of osteoporosis at least one before the index date, a prescription drug fill for HRT or NSAIDs at least one before the index date, and a history of an upper or lower endoscopy at least one year before the index date. The BMI variable was based on the mode of the height and the median weight for all measured heights and weights prior to the year leading up to the index date. BMI was missing for a large proportion of participants (48.68% missing), so we used the BMI data primarily in sensitivity analyses.

### Statistical analysis

We calculated descriptive statistics for the colorectal cancer cases and controls for the covariates of interest. We then created conditional logistic regression models for the association of ever use of bisphosphonates and DDD with colorectal cancer^27^. Models with adjustment for potential confounders, including age, smoking status, alcohol use, CCI, use of NSAIDs, and a previous lower endoscopy were also created. A conditional logistic regression model for each ICD-O-3 site was created with adjustment for potential confounders.

We additionally conducted several sensitivity analyses. We created a conditional logistic regression model which excluded adjustment for previous lower endoscopy. A conditional logistic regression model for only participants with a valid BMI was created and then we separately tested the inclusion of BMI in adjustment. We created a stratified model by duration of membership prior to the index date. The short duration of membership was the first quartile of membership duration (≤4.6 years) and the long duration of membership was the top three quartiles of membership duration (>4.6 years). Sex-stratified conditional logistic regression models were created and we additionally tested the inclusion of adjustment for hormone replacement therapy in the model for females. We created unconditional logistic regression models stratified by previous lower endoscopy and by BMI category. In order to keep all cases and controls in the analysis, we broke the matched pairs and included all mentioned potential confounders and additionally adjusted for the matching covariates including sex, race, region, and years of KPNC membership. We conducted the statistical analyses using SAS 9.3.

## Additional Information

**How to cite this article**: Vogtmann, E. *et al*. Oral bisphosphonates and colorectal cancer. *Sci. Rep.* **7**, 44177; doi: 10.1038/srep44177 (2017).

**Publisher's note:** Springer Nature remains neutral with regard to jurisdictional claims in published maps and institutional affiliations.

## Supplementary Material

Supplementary Tables

## Figures and Tables

**Table 1:** Descriptive characteristics of colorectal cancer cases and controls, Kaiser Permanente, Northern California, 1997–2011.

	Cases		Controls	
	N/Mean	%/SD	N/Mean	%/SD
Number of participants	12,505		599,534	
Ever oral bisphosphonates (>1 yr)	563	4.50%	32,897	5.49%
Ever use oral alendronate (>1 yr)	556	4.45%	32,281	5.38%
Ever use oral etidronate (>1 yr)	13	0.10%	785	0.13%
Ever use oral ibandronate (>1 yr)	4	0.03%	206	0.03%
Ever use oral risedronate (>1 yr)	14	0.11%	989	0.16%
Ever use oral tiludronate (>1 yr)	0	0.00%	4	0.00%
Defined Daily Dose				
Never	11,942	95.50%	566,637	94.51%
<12 months	224	1.79%	11,928	1.99%
> = 12 months	339	2.71%	20,969	3.50%
Age at index	66.40	13.00	65.94	12.90
Female	6,027	48.20%	290,996	48.54%
Race/ethnicity				
Non-Hispanic white	7,519	60.13%	373,257	62.26%
Non-Hispanic black	1,139	9.11%	51,400	8.57%
Hispanic	1,629	13.03%	75,522	12.60%
Other	2,162	17.29%	97,087	16.19%
Missing	56	0.45%	2,268	0.38%
Smoked tobacco (% yes)	5,013	40.09%	214,862	35.84%
Consumed alcohol (% yes)	3,097	24.77%	131,538	21.94%
Body mass index				
Missing	6,286	50.27%	291,630	48.64%
Underweight	17	0.14%	665	0.11%
Normal weight	2,038	16.30%	108,945	18.17%
Overweight	1,859	14.87%	96,253	16.05%
Obese	2,305	18.43%	102,041	17.02%
Charlson comorbidity index	0.85	1.97	0.18	0.79
History of osteoporosis (>1 yr)	5,986	47.87%	308,108	51.39%
Previous lower endoscopy	6,492	51.92%	271,688	45.32%
Previous lower endoscopy (>1 yr)	3,492	27.92%	248,160	41.39%
Previous upper endoscopy (>1 yr)	525	4.20%	37,182	6.20%
Ever use HRT (>1 yr)	2,602	20.81%	146,633	24.46%
Ever use NSAIDs (>1 yr)	8,264	66.09%	427,351	71.28%

Note: >1 yr indicates that the exposure occurred at least one year prior to the index date.

**Table 2:** Association between oral bisphosphonate use and colorectal cancer, Kaiser Permanente, Northern California, 1997–2011.

	Unadjusted		Adjusted	
	OR	(95% CI)	OR	(95% CI)
Ever use oral bisphosphonates	0.77	(0.71, 0.85)	0.82	(0.74, 0.89)
Defined Daily Dose				
Never	Ref		Ref	
<12 months	0.85	(0.74, 0.98)	0.83	(0.72, 0.96)
>= 12 months	0.73	(0.65, 0.82)	0.81	(0.72, 0.90)

Cases and controls were matched on sex, age at time of index date (+/−2 years), duration of membership prior to index date (+/−1 year), race, and region of residence.

The adjusted model additionally included age, smoking, alcohol use, Charlson comorbidity index, use of NSAIDs, and previous lower endoscopy.

**Table 3:** Association between oral bisphosphonate use and colorectal cancer sites, Kaiser Permanente, Northern California, 1997–2011.

	N cases	OR	(95% CI)
Site-specific associations			
Cecum	2,195	0.86	(0.72, 1.03)
Appendix	131	0.19	(0.03, 1.42)
Ascending colon	1,648	0.79	(0.63, 0.99)
Hepatic flexure of colon	541	0.84	(0.57, 1.26)
Transverse colon	830	1.15	(0.86, 1.54)
Splenic flexure of colon	348	0.63	(0.33, 1.21)
Descending colon	547	1.01	(0.64, 1.60)
Sigmoid colon	2,803	0.77	(0.61, 0.96)
Overlapping lesion of colon	61	0.55	(0.15, 2.10)
Colon, NOS	149	0.40	(0.14, 1.15)
Rectosigmoid junction	752	0.66	(0.42, 1.02)
Rectum	2,500	0.73	(0.56, 0.94)

Cases and controls were matched on sex, age at time of index date (+/− 2 years), duration of membership prior to index date (+/− 1 year), race, and region of residence.

The adjusted model additionally included age, smoking, alcohol use, Charlson comorbidity index, use of NSAIDs, and previous lower endoscopy.

**Table 4:** Association between oral bisphosphonate use and colorectal cancer after adjustment for body mass index among participants with measured body mass index, Kaiser Permanente, Northern California, 1997–2011.

	OR without adjustment for BMI	(95% CI)	OR with adjustment for BMI	(95% CI)
Ever use oral bisphosphonates	0.89	(0.80, 0.99)	0.92	(0.83, 1.02)
Defined Daily Dose				
Never	Ref		Ref	
<12 months	0.93	(0.79, 1.09)	0.95	(0.81, 1.12)
>= 12 months	0.87	(0.76, 0.99)	0.90	(0.79, 1.02)

Cases and controls were matched on sex, age at time of index date (+/−2 years), duration of membership prior to index date (+/−1 year), race, and region of residence.

The adjusted model additionally included age, smoking, alcohol use, Charlson comorbidity index, use of NSAIDs, and previous lower endoscopy.

**Table 5:** Sex-specific associations between oral bisphosphonates and colorectal cancer, Kaiser Permanente, Northern California, 1997–2011.

	OR	(95% CI)
Ever use oral bisphosphonates		
Females	0.84	(0.76, 0.92)
Males	0.63	(0.47, 0.85)
*p interaction*	0.0426	
Defined Daily Dose		
Females		
Never	Ref	
<12 months	0.84	(0.73, 0.98)
>= 12 months	0.83	(0.74, 0.94)
Males		
Never	Ref	
<12 months	0.71	(0.47, 1.08)
>= 12 months	0.56	(0.36, 0.86)
*p interaction (<12)*	0.3194	
*p interaction (*>* = 12)*	0.0596	

Cases and controls were matched on sex, age at time of index date (+/−2 years), duration of membership prior to index date (+/−1 year), race, and region of residence.

The adjusted model additionally included age, smoking, alcohol use, Charlson comorbidity index, use of NSAIDs, and previous lower endoscopy.

**Table 6:** Associations between oral bisphosphonates and colorectal cancer stratified by previous lower endoscopy at least one year prior to the index date, Kaiser Permanente, Northern California, 1997–2011.

	OR	(95% CI)
Previous lower endoscopy		
Ever use oral bisphosphonates	0.92	(0.80, 1.04)
Defined Daily Dose		
Never	Ref	
<12 months	0.93	(0.76, 1.15)
>= 12 months	0.91	(0.77, 1.06)
No previous lower endoscopy		
Ever use oral bisphosphonates	0.73	(0.64, 0.83)
Defined Daily Dose		
Never	Ref	
<12 months	0.76	(0.63, 0.91)
>= 12 months	0.71	(0.60, 0.83)

The unconditional logistic regression model adjusted for age, sex, race, region, years of KPNC membership, smoking, alcohol use, Charlson comorbidity index, and use of NSAIDs.
